# Constrictive pericarditis in a post–renal transplant patient: a case report

**DOI:** 10.1186/1752-1947-8-8

**Published:** 2014-01-06

**Authors:** Ranga M Weerakkody, Harshani D Perera, Chaminda Kularathne, Rezvi Sheriff

**Affiliations:** 1 University Medical Unit, National Hospital of Sri Lanka, 01000 Colombo 10, Sri Lanka; 2 Institute of Cardiology, National Hospital of Sri Lanka, 01000 Colombo 10, Sri Lanka; 3 Department of Internal Medicine, Faculty of Medicine, University of Colombo, 00900 Colombo 09, Sri Lanka

**Keywords:** Constrictive pericarditis, Isoniazid prophylaxis, Renal transplant, Tuberculosis

## Abstract

**Introduction:**

Constrictive pericarditis is a rare complication in the post–renal transplant period. It poses a diagnostic dilemma even in the modern era. Its incidence is not known and tuberculosis is implicated in some of the cases.

**Case presentation:**

A 54-year-old Sri Lankan man, in the sixth year of transplant presented with resistant ascites, shortness of breath and elevated creatinine from the baseline. Pre-transplant he was empirically treated for tuberculosis pericarditis and was on isoniazid prophylaxis for 1 year following transplantation. Two-dimensional echocardiography and cardiac catheterization confirmed the diagnosis, and pericardiectomy was performed, which resulted in full resolution of the symptoms as well as the graft function. The histology or bacteriology failed to demonstrate features suggestive of tuberculosis in the surgical specimen.

**Conclusion:**

In constrictive pericarditis, a causative factor is difficult to find. Isoniazid prophylaxis shows benefit in preventing tuberculosis-associated constrictive pericarditis.

## Introduction

Constrictive pericarditis (CP) is a rare post–renal transplant (RT) complication. The incidence of post–RT pericarditis (including non-constrictive types) is 2.4% in cases in which uremia, cytomegalovirus, tuberculosis (TB), non-specific bacterial infection and minoxidil therapy have been described as etiologies [1]. However, one-fourth of the cases have been reported to be idiopathic in etiology. CP still poses a diagnostic dilemma, even in the modern era. A combination of immense morbidity and excellent prognosis following surgery dictates early, accurate diagnosis of the condition. The incidence of post-RT CP is unknown, and only four cases have been reported in the literature [2]. Herein we report a case of a RT recipient with probable tuberculous CP after completion of standard anti-TB treatment and post-RT prophylactic therapy for 1 year.

## Case presentation

A 54-year-old Sri Lankan man who had undergone live related renal transplantation for hemodialysis-dependent, end-stage, hypertensive nephrosclerosis presented to our institution with progressive New York Heart Association class III dyspnea and abdominal distention of several months’ duration. At the time of presentation, he was in the sixth year after transplantation.

His pre-transplant evaluation had revealed refractory serous pericardial effusion despite normal serum albumin levels and intensive hemodialysis. Pericardial fluid taken at that time for acid-fast bacilli (AFB) smear and culture in Löwenstein–Jensen medium was negative, and there was no proof of TB elsewhere. On the basis of a strongly positive tuberculin skin test and the patient’s symptoms, however, 6 months of a standard four-drug anti-TB regimen was commenced, which led to an excellent response prior to the transplantation procedure.

After the transplant, the patient had been started on routine immunosuppressive therapy with prednisolone, cyclosporine and mycophenolate mofetil. In the first 6 years following transplant, he achieved a good quality of life with a baseline creatinine level of 120μmol/L.

Upon the patient’s presentation to our institution, his physical examination revealed engorged jugular veins in the neck, tense ascites with tender hepatomegaly, as well as mild bilateral pitting edema of the ankles. His heart sounds were normal, with no murmurs or added sounds. His respiratory system was clinically normal.

The patient’s chest X-ray showed normal lung fields with a small heart shadow and no visible calcifications. Echocardiography revealed biatrial enlargement, left atrial diameter of 46mm, normal-size ventricles, good left ventricular function and a dilated inferior vena cava (27mm) with flow reversal. Paracentesis revealed the presence of transudate fluid. Subsequent cardiac catheterization showed early, rapid diastolic filling and equalization of pressure between the ventricles consistent with CP (Table 1). The patient’s serum creatinine level was 212μmol/L at presentation.

**Table 1:** **Results obtained from pressure study during cardiac catheterization**^**a**^

**Chamber**	**Pressure (mmHg)**
RA	22
RV	40/15
RVEDP	23
MPA	36/20 (mean = 28)
LV	95
LVEDP	23
Aorta	105/65

^a^LV, Left ventricle; LVEDP, Left ventricular end-diastolic pressure; MPA, main pulmonary artery; RA, Right atrium; RV, Right ventricle; RVEDP, Right ventricular end-diastolic pressure.

**Figure 1:**
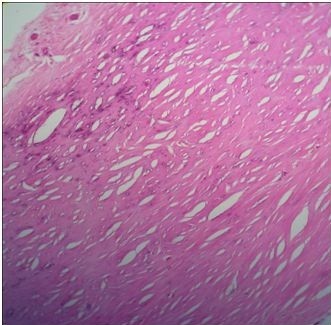
**Hematoxylin and eosin–stained pericardial tissue section showing no evidence of granulomatous inflammation.** Foci of microcalcifications are present in fibrous tissue. Original magnification, ×40.

He underwent an elective, uncomplicated total pericardiectomy, which provided symptomatic relief. Macroscopy revealed a very thick pericardium up to 15mm in some areas, causing constriction of all chambers of the heart. Histology showed a thick layer of fibrous tissue with early calcifications, but no granulomas or AFB (Figure 1). Analysis of pericardial tissue for *Mycobacterium tuberculosis*, including Ziehl–Neelsen stain, culture on Löwenstein–Jensen medium and polymerase chain reaction, was negative.

He was started on lifelong prophylactic isoniazid therapy. A follow-up echocardiogram taken 1 month after surgery was normal. At the 3-month post-operative examination, he had no significant symptoms and was able to walk briskly for 30 minutes on a flat surface. His graft function had improved markedly, and his serum creatinine level had dropped to 144μmol/L.

## Discussion

CP occurs when a thickened fibrotic pericardium, of whatever cause, impedes normal diastolic filling. Acute and subacute forms of pericarditis may deposit fibrin, which in turn can evoke pericardial effusion. This often leads to pericardial inflammation, chronic fibrotic scarring, calcification and restricted cardiac filling [3]. There are no data on Sri Lankan patients regarding the etiology of CP, but Indian data show that TB is the causative factor in two-thirds of the cases [2]. Classically, dry, effusive, absorptive and constrictive stages in the pathology of CP have been described, and patients may present with symptoms at any of these stages. The sensitivity of pericardial biopsy for demonstrating TB ranges from 10% to 64%. Given the wide Bacillus Calmette–Guérin vaccination, cross-reactivity with environmental mycobacteria and use of immunosuppressive agents, tuberculin skin tests have become less reliable as a diagnostic tool in post-RT patients. Interferon γ testing (QuantiFERON®-TB; Cellestis, Chadstone, Australia) is much too expensive for use with most of the patients in Sri Lanka. Guidelines do exist for the diagnosis of tuberculous CP [2], but the validity of them for a medium-endemicity country such as Sri Lanka has not been established. Other causes of CP include acute or relapsing viral or idiopathic pericarditis, trauma, cardiac surgery, mediastinal irradiation, purulent infection, histoplasmosis, neoplastic disease, connective tissue disorders and chronic hemodialysis [1]. Pericardiectomy is recommended in cases of deterioration hemodynamics or in association with pericardial calcification. Mortality following pericardiectomy in patients with tuberculous PC ranges from 3% to 16% [2].

We were able to identify only four case reports in the literature describing CP following renal transplantation. Two of the patients presented 4 years after RT, and another one presented 4 months after RT with symptoms of right heart failure. The fourth was asymptomatic, and CP was identified during routine Doppler echocardiography and subsequently confirmed with right heart catheterization [2]. The etiology of CP was not established in any of these four cases. The absence of pericarditis prior to RT makes uremic etiology unlikely. The most likely etiology is TB.

There are marked discrepancies among centers regarding the success of prophylaxis of TB in post-RT patients [4]. There is ample evidence to suggest that isoniazid prophylaxis is protective against the development of post-RT TB [5,6]. In most of the studies reported, isoniazid 100mg daily was used for 1 year, which was shown to be beneficial, but data on long-term follow-up are lacking. Isoniazid at prophylactic dosages has been safe: Transient hyperbilirubinemia has been reported in less than 0.5% of the patients [6]. Unfortunately, our patient developed his disease more than 6 years post-RT after 1 year of prophylactic isoniazid therapy, raising concerns about whether the duration of prophylaxis is in fact enough. Further studies are needed to establish the duration and adequacy of TB prophylaxis.

## Conclusion

No clear etiology can be found for CP after renal transplantation. A significant number of these cases are due to TB, in the setting of which its diagnosis is a challenge. Isoniazid prophylaxis has shown good results in prevention; as our present case shows, however, tuberculous CP can occur even after 1 year of isoniazid prophylaxis. Hence, the exact dose and duration of isoniazid prophylaxis are areas requiring further research.

## Consent

Written informed consent was obtained from the patient for publication of this case report and any accompanying images. A copy of the written consent is available for review by the Editor-in-Chief of this journal.

## Abbreviations

AFB: Acid-fast bacilli; CP: Constrictive pericarditis; LV: Left ventricle; RT: Renal transplant; TB: Tuberculosis.

## Competing interests

The authors declare that they have no competing interests.

## Authors’ contributions

RMW was primarily involved in the management of the nephrological issues of the patient. RMW and NJAHDP wrote the first manuscript draft and surveyed the literature. CK performed the cardiologic investigations and offered opinions regarding cardiologic management. MHRS is the supervising consultant and gave final authorization for publication of the manuscript. All authors read and approved the final manuscript for publication.
